# The Effect of Pentoxifylline on *bcl-2* Gene Expression Changes in Hippocampus after Ischemia-Reperfusion in Wistar Rats by a Quatitative RT-PCR Method 

**Published:** 2013

**Authors:** Soyar Sari, Mehrdad Hashemi, Reza Mahdian, Kazem Parivar, Mehdi Rezayat

**Affiliations:** a *Department of Biology, Islamic Azad University, Science and Research Branch Tehran, Iran.*; b *Department of Genetics, Islamic Azad University, Tehran Medical Branch.*; c *Biotechnology Research Centre, Molecular Medicine Department, Pasteur Institute of Iran, Tehran, IR Iran.*; d *Department of Pharmacology, School of Medicine, Tehran University of Medical Sciences, Tehran, Iran. *

**Keywords:** Ischemia-reperfusion, Apoptosis, *bcl-2*, Real-time PCR

## Abstract

Ischemia-reperfusion injury is the tissue damage caused when blood supply returns to the tissue after a period of ischemia or lack of oxygen. Ischemia-reperfusion brain injury initiates an inflammatory response involving the expression of adhesion molecules and cytokines. Twenty–four male Wistar rats (250-300 g body wt) were used in this study. The animals were divided into four groups of 6 rats each: I: Control group that was subjected to ischemia-reperfusion, II: Ischemia-reperfusion group that was subjected to all surgical procedures, III: Drug group that received pentoxifylline (200, 400 and 600 mg/kg) 60 min before and after ischemia and IV: Vehicle group that received saline. Seventy two h after ischemia-reperfusion, the hippocampus was taken for studying the changes in *bcl-2 *gene expression. We used quantitative real-time PCR for the detection of *bcl-2 *gene expression in ischemia and drug groups and then compared them to normal samples. The results showed the gene dosage ratio of 0.66 and 1.5 for ischemia group and the drug groups, respectively. The results also showed the *bcl-2 *gene expression declined in ischemia group as compared to the drug group. Furthermore, we observed a significant difference in the *bcl-2 *gene expression between ischemia and drug groups. These findings are consistent with anti-apoptotic properties of *bcl-2 *gene. Furthermore this method provides a powerful tool for the investigators to study brain ischemia and respond to the treatment drugs with anti-apoptotic agents.

## Introduction

Ischemia-reperfusion brain injury initiates an inflammatory response involving the expression of adhesion molecules and cytokines (1). Ischemia-reperfusion injury is the tissue damage caused when blood supply returns to the tissue after a period of ischemia or lack of oxygen (2, 3). The absence of oxygen and nutrients from blood during the ischemic period creates a condition in which the restoration of circulation results in inflammation and oxidative damage through the induction of oxidative stress rather than restoration of normal function (4). The inflammatory response partially mediates the damage of reperfusion injury. White blood cells carried to the damaged area by the newly returning blood release inflammatory factors such as interleukins and tumor necrosis factor (TNF-*α*) (5-9). TNF-*α *stimulates the production of the *bcl-2 *family members from the cytoplasm to the outer mitochondrial membrane (10-13). This leads to mitochondrial swelling and induces apoptosis (14, 15). Therefore, pharmacological agents could decrease the production of TNF-*α *in the process of ischemia-reperfusion injury that results in reduced reperfusion injury, Pentoxifylline (PTX) is a drug that has multiple properties. It decreases oxygen and the production of free radicals (16) and inhibits TNF-*α *in mononuclear phagocytes. A study indicated that PTX has some protecting effects on remote kidney injury only in the early phase of reperfusion due to ischemia-reperfusion injury (17), however, another study indicated that PTX decreases oxidative damage in rat liver after ischemia-reperfusion (18). In this study, we have designed and optimized quantitative real-time PCR assay based on SYBR Green I chemistry to determine the effect of PTX on *bcl-2 *gene expression changes in hippocampus after ischemia-reperfusion injury in rat. 

## Experimental

*Animals*

Twenty-four male Wistar rats (250-300 g body wt) were used in this study. The animals were maintained on the basal diet and water, and then anesthetized by intraperitoneal injection of pentobarbital sodium (40 mg/kg). Their body temperature was kept between 36 and 37 °C by a heating lamp. Carotid artery was closed by a vascular clamp for 20 min. After the period of ischemia, the clamp was removed and the hippocampus was reperfused. The rats were divided into four groups (n = 6 in each group) ; I: Control group that was subjected to ischemia-reperfusion, II: Ischemia-reperfusion group that was subjected to all surgical procedures, III: Drug group that received pentoxifylline (200, 400 and 600 mg/kg ) 60 min before and after ischemia and V: Vehicle that received saline (19). Seventy two h after the ischemia-reperfusion, the hippocampus was taken for studying the changes in *bcl-2 *gene expression. After this time of injury, the rats were euthanized and the brain tissue (about 100 mg) was immediately collected from rat’s hippocampus. 

*RNA isolation, DNA digestion and reverse transcription*

The tissue samples were treated with total RNA isolation reagent (Sigma) as recommended by the manufacturer and the extracted RNA was purified using RNeasy Mini Kit (Qiagen). The concentration and purity of the purified RNA were determined by spectrophotometry. High quality RNAs (A260/280≥1.8) were selected and kept at -80 °C until use for cDNA synthesis. Up to 1 μg RNA was converted to cDNA by using QuanTitect® Reverse Transcription Kit (Qiagen) according to the manufacturer›s instruction. To verify the integrity of the cDNA, a PCR experiment was performed using glyceraldehydes-3-phosphate dehydrogenase (GAPDH) specific primer. The primers for real-time PCR of *bcl-2 *and *GAPDH *gene expression were designed by the Primer Express v.3.0 software (Applied Biosystems, Foster City, USA). 

*Real-time PCR with SYBR green I*

The selected primers underwent an extensive search using BLAST tool (www.ncbi.nlm.nih.gov/blast). The characteristics of the primers used in this study have been summarized in Table 1.

**Table 1:** Characteristics of the primers used in the real-time PCR assay

rat-*bcl2*-F	ATCGCTCTGTGGATGACTGAGTAC
rat-*bcl2*-R	AGAGACAGCCAGGAGAAATCAAAC
rat-*GAPDH*-F	AAGTTCAACGGCACAGTCAAGG
rat-*GAPDH*-R	CATACTCAGCACCAGCATCACC

 Real-time PCR was carried out in optical grade 96-well plates (Micro amp, Applied Bio systems, Singapore) at reaction volume of 25 mL, including 12.5 SYBR Green Master Mix (primer design), 300 nM primer and 5 ng genomic template DNA. All samples were run in duplicate. Thermal cycling was performed on the Applied Biosystems 7300 real-time PCR system using the following cycling conditions: 95°C for 10 min, and 40 cycles at 95°C for 15 sec, and 60°C for 1 min. Each complete amplification stage was followed by a dissociation stage at 95°C for 15 sec and 60 °C for 30 sec. Then, the temperature was ramped up from 60°C to 95 °C ( 0.03°C/s), and fluorescence intensity data was collected continuously over the ramping stage for 20 min. Melting curve analysis was performed according to the dissociation stage data and reactions with a single peak at expected temperature melting (Tm) were considered for further analysis.

*Data analysis*

Quantitative analysis was performed by the measurement of threshold cycle (CT) values during the exponential phase of amplification. The parameter CT was defined as the cycle number at which the amplification plot passed a fixed threshold. In each assay, mCT was the mean CT value of duplicate amplifications. Relative quantity of *bcl-2 *gene was determined using comparative CT method and ΔCT was calculated as the difference between the CT values of the *bcl-2 *and the CT value of *GAPDH *gene. The data were analyzed using the following formula: Gene dosage ratio = 2^−ΔΔCT^, where −ΔΔCT = [mCT *bcl-2 *(normal sample) − mCT *GAPDH *gene (normal sample)] − [mCT *bcl-2 *(test sample) – mCT *GAPDH *gene (test sample) (21). Gene dosage ratios were relative to the mean ΔCT value of these samples. Data processing was analyzed with ABI Prism 7300 Sequence Detection System (version 1.2.3, Applied Biosystems, UK). The graph preparation was performed using Microsoft Excel 2007 and RJS Graph 3.90.10 (20).

## Results

**Figure 1:**
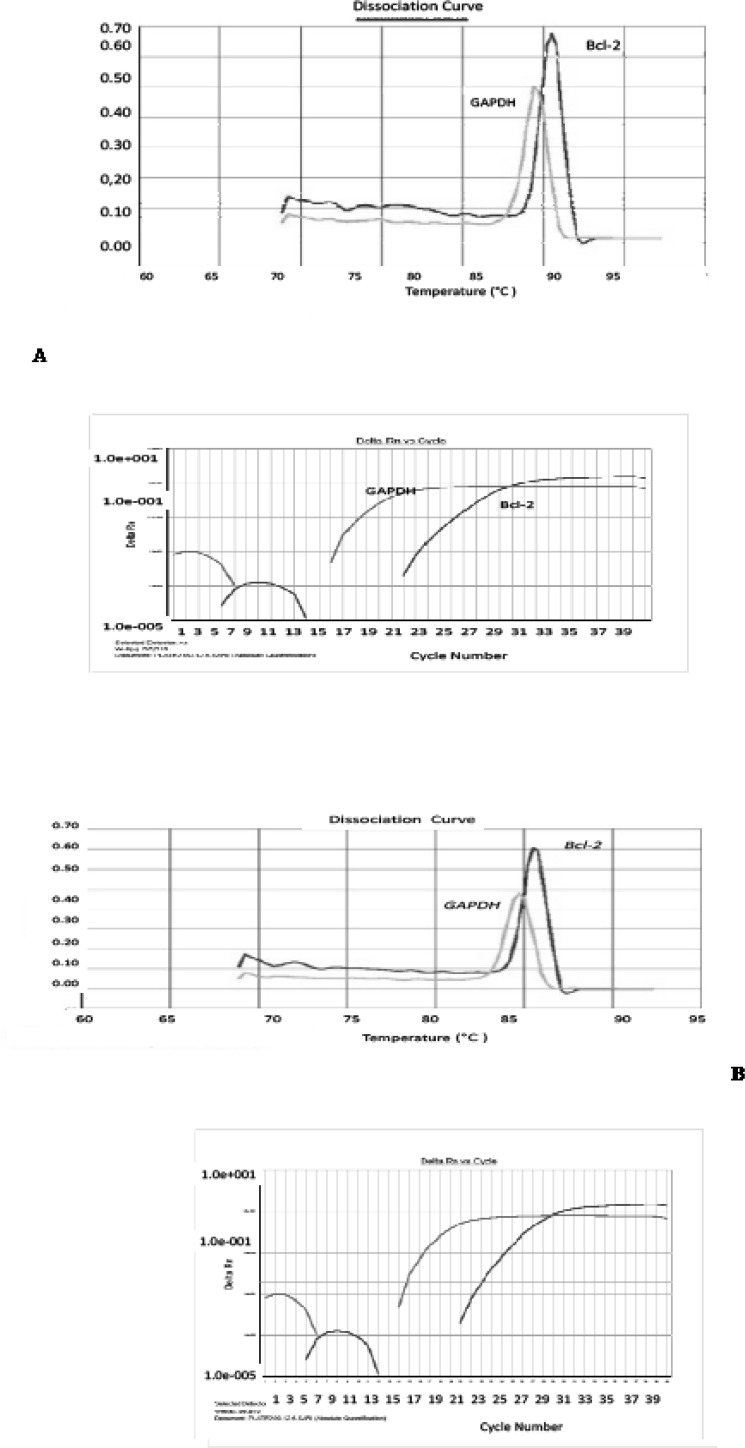
Amplification and melting curve analysis of real-time PCR for *bcl-2 *and *GAPDH *genes. (A) Melting curve analysis for PCR products obtained with the specific primer pairs for *bcl-2*, and *GAPDH *genes in ischemia sample. Amplification curve analysis of *bcl-2 *and *GAPDH *genes in (B) PTX group, (C) normal group and (D) vehicle group

Pentoxifylline effects on *bcl-2 *gene expression changes in hippocampus after ischemia-reperfusion were compared with the ischemia group. Using this method, tested and normal samples were analyzed. As expected, there was a significant difference between the tested and normal samples in multiplex PCR. To optimize and validate the real-time PCR assay before using ΔΔCT method for gene expression, a validation experiment was performed to determine the PCR efficiencies of the target and the reference genes. The input amount of template DNA was plotted against the corresponding CT values. The slope of the best-fit lines were within the acceptable range of −3.6<slope<−3.1 (http://www.gene-quantification.de). The consistency of all the PCR reactions through a wide range of template DNA concentrations (3–50 ng) was assessed by plotting ΔCT values of *bcl-2 *gene against the input amount of DNA. The absolute slopes of the best-fit lines were ≤0.1 for *bcl-2 *gene which indicated the validity of the ΔΔCT relative quantitation. Melting curve analysis was performed for every single reaction to exclude the amplification of non-specific products (Figure 1).

**Figure 2:**
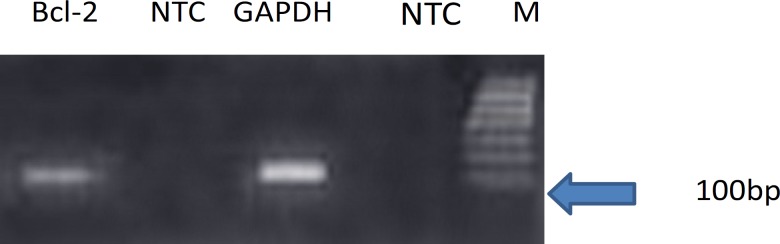
Results of Real-time PCR analysis for expression of *bcl-2 *gene. *bcl-2 *and *GAPDH. *NTC: non-template control. M: DNA Size marker

 Each valid amplification reaction displayed a single peak at expected Tm. Furthermore, gel electrophoresis analysis of the PCR products revealed a single band with the expected size for each amplicon, (Figure 2). The results showed the gene dosage ratio of 0.66 for ischemia group and 1.15 for drug group. The results also showed the *bcl-2 *gene expression declined in ischemia group as compared to the drug group. 

## Discussion

Ischemia-reperfusion injury is the tissue damage caused when blood supply returns to the tissue after a period of ischemia or lack of oxygen. Ischemia-reperfusion brain injury initiates an inflammatory response involving the expression of adhesion molecules and cytokines. The aim of this study was the effect of pentoxifylline on *bcl-2 *gene expression changes in hippocampus after ischemia-reperfusion in Wistar rats. We designed and optimized quantitative real-time PCR assay using SYBR green I technologies from Applied Biosystems. We selected *bcl-2 *gene, because *bcl-2 *family of proteins functions as pro- and anti-apoptotic members (21). *bcl-2 *members such as *bax, bak, bad or bcl-Xs *promote apoptosis, whereas other members such as *bcl-2 *and *bcl-Xl *prevent apoptosis by blocking the translocation of cytochrome c, and subsequent caspase activation. Mitochondria are involved in excitotoxic injury during cerebral ischemia and the release of cytochrome c, an apoptogenic factor that propagates death signals by triggering caspases leading to cell death. Using these assay, status of all subjects was successfully determined. We expanded the coverage of the detectable *bcl-2 *gene by SYBR Green assay for *bcl-2 *gene expression. This issue could be increased in the drug group and decreased in ischemia group. Honkaniemi *et al*. (22) indicated that in ischemia group the *bcl- 2 *gene was decreased after 72 h. Prakasa and Yoshida (23) showed that in ischemia group the *bcl-2 *gene was decreased after 24 h, but *bax *expression was increased. The results of SYBR Green method were similar to those of obtained from the immunohistochemical analysis and TUNEL assay (24). In the present study, the mean value of the ratios, obtained from tested and normal samples using SYBR Green assay for *bcl-2 *gene, was in agreement with the results reported by Honkaniemi *et al*. ( 22) and Prakasa (23). 

## Conclusion

This work represents the first systematic study of the simultaneous changes in *bcl-2 *gene expression for transient brain ischemia in Wistar rats using real-time PCR technique. Although many studies have focused on *bcl-2 *mRNA expression in ischemic brain injury, conventional techniques such as Northern blot analysis, quantitative RT-PCR and *in-situ *hybridization are not able to detect small amounts of mRNA, and do not provide quantitative measure of the amounts of mRNA present in the samples. The real-time PCR requires a very small amount of mRNA. In the present study, *bcl-2 *promoted the transcription of mRNA in the drug group, but prevented the transcription of mRNA in the ischemia group. In addition, *bcl-2 *mRNA expression significantly declined in the ischemia group as compared to drug group. These findings are consistent with anti-apoptotic properties of *bcl-2 *gene. Furthermore this method provides a powerful tool for the investigators to study brain ischemia and respond to the treatment drugs with anti-apoptotic agents. 
